# Thalamic disconnection from prefrontal cognitive control networks contributes to thalamic aphasia

**DOI:** 10.1093/braincomms/fcaf191

**Published:** 2025-05-16

**Authors:** Anika Stockert, Sophia Hormig-Rauber, Julian Klingbeil, Sophie Marie Meixensberger, Karl-Titus Hoffmann, Dorothee Saur, Max Wawrzyniak

**Affiliations:** Neuroimaging Laboratory, Department of Neurology, Leipzig University, Medical Center, 04103 Leipzig, Germany; Neuroimaging Laboratory, Department of Neurology, Leipzig University, Medical Center, 04103 Leipzig, Germany; Neuroimaging Laboratory, Department of Neurology, Leipzig University, Medical Center, 04103 Leipzig, Germany; Department of Neurology, Halle University, Medical Center, 06120 Halle, Germany; Neuroimaging Laboratory, Department of Neurology, Leipzig University, Medical Center, 04103 Leipzig, Germany; Institute of Neuroradiology, Leipzig University, Medical Center, 04103 Leipzig, Germany; Neuroimaging Laboratory, Department of Neurology, Leipzig University, Medical Center, 04103 Leipzig, Germany; Neuroimaging Laboratory, Department of Neurology, Leipzig University, Medical Center, 04103 Leipzig, Germany

**Keywords:** thalamus, language, aphasia, structural connectivity, disconnection mapping

## Abstract

Language impairments after thalamic lesions, referred to as thalamic aphasia, underscore a subcortical involvement in language processing. In this study, we investigated how the thalamus structurally connects to the cortex to support language functions. Our hypothesis posits that disconnection of white matter tracts between the left thalamus and regions of left hemisphere language and cognitive control networks, such as prefrontal, inferior frontal, and temporal cortices, are associated with thalamic aphasia. We employed a non-parametric lesion-network mapping approach in a retrospective cohort of patients with first-ever thalamic stroke. This method enables the identification of structural disconnections that disrupt signal transmission along white matter fibre pathways, subsequently impairing processing within brain networks. To investigate potential associations between disconnection patterns and thalamic aphasia, we individually mapped fibre tracts affected by the thalamic stroke lesions using diffusion-weighted normative structural connectome data. Statistical comparisons were then made between disconnection maps of patients with and without language impairments. The study encompassed 101 patients, with a mean age of 64.1 years (standard deviation, 14.6), including 57 patients with left-sided, 42 with right-sided, and 2 with bilateral thalamic lesions. We observed that language impairments were linked to disconnection of fibres in the left anterior limb of the internal capsule. These fibres constitute a pathway within the anterior thalamic radiation, connecting the mediodorsal thalamus to a region in the left dorsomedial prefrontal cortex. An additional exploratory analysis revealed functional connectivity between this cortical area and the left hemisphere’s language-related inferior frontal and lateral temporal cortices. Meanwhile, we found no evidence for direct structural disconnection between the thalamus and left inferior frontal or temporal cortices.

Interrupted signalling along the left anterior thalamic radiation to the left dorsomedial prefrontal cortex is a potential mechanism underlying thalamic aphasia. Given its significance for the subcortical involvement in language processing, our findings suggest a plausible path for thalamic signals to participate in the interplay between networks for cognitive control and language processing.

## Teaser

In a cohort of thalamic stroke, Stockert *et al*. investigated how the thalamus structurally connects to the cortex to support language. They report that language impairments are related to left anterior internal capsule prefrontal cortex disconnection. They conclude that disrupted signalling along this pathway is one mechanism underlying thalamic aphasia.

## Graphical Abstract

**Graphical Abstract:**
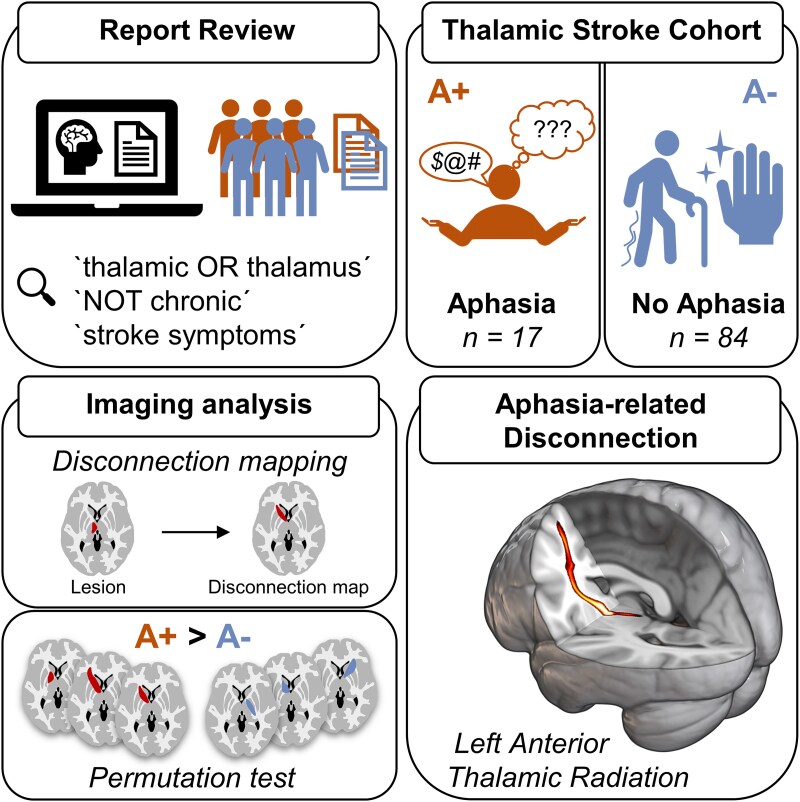

## Introduction

Language is traditionally viewed as a cortical function accomplished primarily by the integration within a domain-specific left-lateralized inferior frontal and lateral temporal network.^1^ Prefrontal, cingulate, and parietal cortices involved in domain-general computations (e.g. cognitive control and attention) relevant to multiple cognitive functions have been suggested to facilitate language.^2,3^ In addition, an emerging body of research advocates that circuits between cortical and subcortical regions collectively contribute to cognitive functions in general^4^ and to language in particular.^5-9^ Because direct evidence that left-sided subcortical lesions cause language impairments (i.e. subcortical aphasia) has so far come from smaller samples or reviews of historic cases with variable lesion locations,^10-12^ it is still unclear how these lesions affect networks involved in language. While some of the subcortical aphasia syndromes have been attributed to cortical hypoperfusion in language networks lying in the same vascular territory as a subcortical lesion,^13^ this concept does not account for language impairments following thalamic lesions that are in the territory of the posterior cerebral artery. Alternative hypotheses therefore take into account that cortical dysfunction could be caused by functional disconnection, leading to disrupted signalling between subcortical and cortical regions of the dominant (left) hemisphere.^9,12^

In a previous retrospective study,^9^ we showed that stroke lesions to the left mediodorsal thalamic nucleus are associated with language impairments. Moreover, thalamic lesions causing aphasia were functionally connected to left hemisphere fronto-temporal language networks and to bilateral domain-general networks. This network affection was confirmed by a later prospective study.^14^ We argued that in thalamic aphasia, efficient integration between these left-lateralized networks is impaired because left-sided thalamic nuclei are functionally disconnected. In the current study, we explore the structural foundations of a thalamic involvement in language in these cortico-thalamo-cortical networks.

As one principle of organization, the thalamus builds bidirectional cortico-thalamo-cortical circuits with direct axonal projections to and from the ipsilateral neocortex. Thalamic nuclei that follow this connectivity pattern are referred to as higher-order nuclei, for example, the pulvinar, mediodorsal, and anterior thalamic nuclei.^15^ In humans, brain structural connectivity can be measured *in vivo* using diffusion-weighted imaging MRI data. Structural connectivity describes the macroscopic anatomical organization by means of fibre tracts that allow for direct signalling between regions. For higher-order thalamic nuclei, structural connectivity has been described with cortical regions involved in cognitive functions in general and language in particular. The lateral temporal and parietal cortices mainly connect with the pulvinar and mediodorsal nucleus, the cingulate cortex connects with the anterior nuclei, whereas the medial and lateral prefrontal cortices principally connect with the mediodorsal nucleus.^16^ Importantly, in these studies, structural connectivity was derived by parcellating cortical grey matter according to anatomical landmarks unrelated to function.

To better understand the relationship between thalamic connectivity and language function, we employ a method known as structural disconnection mapping, which allows us to directly link disturbed neural connectivity to language impairments. This approach is based on the observation that not only focal damage to eloquent grey matter regions but also disconnections between interconnected brain regions that jointly contribute to a function cause impairments.^17,18^ Here, disconnection implies that interrupted signalling along white matter fibre pathways results in impaired processing between regions. Disconnection mapping is performed using normative structural connectome data from healthy subjects. Individual white matter pathway disruption is estimated by seeding fibre tracking from individual lesions. The identification of pathways associated with a behavioural impairment is achieved by statistically comparing these disconnection maps between patients with and without a certain symptom.

In this retrospective observational study of patients with thalamic stroke, we investigated the structural basis of thalamic involvement in language processing. We applied structural disconnection to symptom mapping to a recently published dataset of 101 patients with thalamic stroke.^9^ By directly comparing structural disconnection patterns associated with language impairments against a baseline that includes other symptoms, we aimed to identify white matter fire tracts implicated in thalamic aphasia. We hypothesized that a distinct pattern of thalamic structural connectivity with left hemisphere regions that contribute to both general cognitive functions and language processing would be associated with language impairments. In this way, the analysis contributes to the discussion of how the thalamus is structurally integrated into different networks critical for language processing.

## Materials and methods

### Participants

All analyses were based on lesion and behavioural data of 101 patients (64.1 ± 14.6 years; mean ± SD, 40 females; 96 right-handed) with first-ever ischaemic thalamic strokes, of whom 17 presented with language impairments. The same 101 patients were analysed previously with voxel-based lesion-symptom mapping and functional lesion-network mapping.^9^

Patients with thalamic strokes were identified retrospectively using an automated review of radiology reports with the keywords ‘Thalamus’ or ‘thalamisch’ (engl. thalamic) or ‘thalamo’. Radiology and medical reports were then reviewed for the following criteria: Inclusion criteria were acute or subacute ischaemic (symptom-onset within < 2 weeks) first-ever thalamic stroke in patients aged 18 years or older. Exclusion criteria were radiologic description of chronic, non-ischaemic (e.g. haemorrhage, tumour or metastasis), or previous other stroke lesions (in any vascular territory), major microvascular brain damage (Fazekas scale > 2) or relevant brain atrophy. Furthermore, patients with other pre-existing neurologic disorders affecting the CNS (e.g. dementia and Parkinson’s disease) were excluded.

### Behavioural assessment

Symptoms were assessed retrospectively based on the complete medical report including medical history, examination in the emergency room, and repeated examinations during treatment on the stroke unit. Additionally, patients were evaluated at least once by a trained speech and language therapist within 24 h of stroke unit admission. If aphasia was suspected, a standardized test (Aphasia Check List)^19^ was performed by speech and language therapists. Because of the sometimes subtle and transient symptoms, language impairments were considered present in patients with a medical history or clinical documentation of new-onset impaired communicative abilities, including reduced fluency, spontaneous speech or word-finding difficulties, paraphasias, neologisms, lexical-semantic deficits, problems during naming or repetition, and impaired comprehension or reading. Based on the treating physicians', physical or occupational therapists’ evaluation, motor deficits included all documented new-onset disorders of movement (i.e. altered muscle tone, impairment of coordination, standing, gait or muscle strength) in at least one body region. Similarly, sensory deficits were considered present in the case of new-onset unilateral abnormalities in touch, pain, or temperature sensation and reported paresthaesia in at least one body region. Dysarthria included slurred or unintelligible speech.

### Lesion delineation

Stroke lesions were manually delineated on routine CT (*n* = 5) or MRI (*n* = 96) scans using MRIcron (https://www.nitrc.org/projects/mricron/). Spatial normalization of the lesion masks to the MNI152 space (isotropic voxels of 1 × 1 × 1 mm) was performed with the Clinical Toolbox for SPM12 (v7487, Wellcome Trust Centre for Neuroimaging, London, United Kingdom) using cost function masking.^20^

### Disconnection mapping

Structural disconnection was estimated based on normative connectome data of 25 healthy participants aged 49–64 years (10 females and 15 males) from the Enhanced Nathan Kline Institute (NKI) Rockland sample.^21^ We have demonstrated in a prior study that the size of n = 25 provides a reasonable balance between accuracy and computation time.^18^ We used diffusion-weighted MRI scans (2 × 2 × 2 mm, 128 directions, *b*-value 1500 s/mm², and additional nine b0-weighted images, anterior–posterior phase encoding) for probabilistic fibre tracking and T1-weighted magnetization prepared rapid gradient-echo images (1 × 1 × 1 mm, repetition time/echo time: 1900/2.52 ms, flip angle: 9°) for spatial normalization.

To test for associations between structural disconnection and language impairments, we mapped fibre tracts affected by the stroke lesions on an individual basis using normative structural connectome data. Preprocessing of the normative diffusion-weighted data as well as probabilistic fibre tracking was performed with the FMRI Software Library (FSL) 6.0^22^ as previously described in a methodological validation of voxelwise structural disconnection mapping.^18^ In short, we performed motion and eddy-current correction (eddy_correct), brain extraction (bet2), Bayesian estimation of diffusion parameters obtained using sampling techniques with crossing fibres (bedpostx) and probabilistic fibre tracking (probtrackx2) seeded from the individual lesion map. Unfortunately, it was technically not possible to correct warping artefacts because the data did not include B0 images with different phase encoding directions. The seed region was spatially restricted to a bilateral thalamus mask to account for lesions partly located outside of the thalamus (e.g. due to spatial inaccuracy caused by the normalization process). To adjust for different lesion sizes and effects of distance, resulting path distributions were corrected for the length of the pathways (−pd), spatially normalized (flirt and fnirt) to MNI152 space, and divided by the total number of generated fibres. This procedure resulted in 25 path distribution maps for each of the 101 stroke lesions. These 25 maps per patient were then averaged, and the result was binarized with an optimal threshold of > 0.15.^18^ The resulting 101 individual binary disconnection maps represented fibre tracts affected by the respective stroke lesions.

### Statistical analyses

To examine potential behavioural confounders, we assessed the independence of all symptoms (i.e. aphasia, dysarthria, left- and right-sided disorders of movement, and sensory symptoms) using one-tailed Fisher's exact tests, specifically testing for positive associations.

To map disconnection to symptoms on the group level, we tested for differences between the disconnection maps of patients with (*n* = 17) and without (n = 84) language impairments. All second-level analyses were performed using Matlab (Mathworks, USA) and modified niiStat (https://www.nitrc.org/projects/niistat/) core functions. We utilized a mass-univariate approach within the framework of the general linear model using lesion size as a covariate of no interest. To increase anatomical validity, only voxels with sufficient disconnection affection (i.e. voxels disconnected in at least five patients) were analysed. Significance was assessed by 5000 random permutations of the design matrix using Freedman-Lane procedure^23^ to obtain the critical threshold corresponding to *P*(FWE) < 0.05 on the voxel-level.^24^

To account for potential behavioural confounders, we conducted an additional specificity analysis. Behavioural variables that did not demonstrate statistical independence from aphasia in Fisher's exact tests were included as additional covariates of no interest.

Since the main result was located in the white matter of the anterior limb of the internal capsule, we performed two additional analyses. First, another probabilistic fibre tracking was used to reconstruct a complete tract and to identify the cortical areas it connects to. Because fibre tracts are the basis for signalling between regions of grey matter, this analysis was aimed at determining which areas were likely to show dysfunction due to structural disconnection. We employed a similar approach to the disconnection mapping described earlier, utilizing group-level significant voxels from the second-level analysis as seed regions. The result was thresholded (instead of binarized) with the same threshold of > 0.15. Second, because probabilistic fibre tracking is unable to map an entire network of multiple and polysynaptically connected regions, we performed another exploratory analysis. In this analysis, we mapped the functional network associated with the cortical grey matter region where fibres associated with language impairments were connected. We defined a 6-mm radius sphere around the coordinate where the probabilistic fibre tracking terminated (MNI coordinate in mm: *x* = −13, *y* = 23, and *z* = 62). This served as the seed for a functional connectivity analysis based on resting-state fMRI data of a normative sample of 65 healthy subjects from the publicly available Enhanced NKI-Rockland sample.^21^ For a better comparability, we used the same sample and procedures as described in our previous publication on thalamic aphasia.^9^

To support the specificity of the results, we repeated all analyses contrasting thalamic disconnection maps of patients with and without right-sided disorders of movement.

All reconstructed tracts and adjacent grey matter were identified based on the Johns Hopkins University (JHU) atlas distributed with MRIcron.

### Standard protocol approvals and patient consents

In compliance with the laws and regulations of the Federal State of Saxony, this retrospective analysis of a published data set did not require an ethics committee approval. On the legal basis of the University of Leipzig Medical Center admission contract, patients or their legal guardians gave written consent to the storage of all medical data. By law (§34 Sächsisches Krankenhausgesetz), physicians are allowed to process medical data stored within their institution for scientific purposes.

## Results

### Demographics and clinical characteristics

We analysed data from 101 patients (64.1 ± 14.6 years; mean ± SD, 40 females, 96 right-handed) with thalamic stroke. Fifty-seven patients had left, 42 patients had right, and 2 patients had bilateral thalamic lesions. Seventeen patients (all with left-sided thalamic lesions) presented with language impairments. The most commonly reported impairments were word-finding difficulties and reduced spontaneous speech. Deficits like impaired comprehension, paraphasias, and naming difficulties were considerably less frequent. All symptoms reported can be found in Table 1 in the original publication of the patient cohort.^9^ Forty-four patients presented with right-sided and 32 patients with left-sided disorders of movement. Thirty-four patients suffered from right and 37 from left sensory deficits. Forty-eight patients presented with dysarthria. We found dependence between aphasia and right-sided disorders of movement as well as between disorders of movement and sensory symptoms for both sides (Fisher’s exact tests, *P* < 0.05, see Supplementary Table 1).

**Table 1:** Aphasia-related disconnection

Cluster size *k*	Peak *t* value	Peak coordinate (*x*, *y*, *z* in mm)	Anatomical label (JHU atlas)
119	5.86	−22, 2, 15	Anterior limb of internal capsule
3	6.05	−5, −24, −2	Midbrain
2	5.78	−22, 8, 14	Anterior limb of internal capsule
2	5.85	−14, −4, 7	Knee/posterior limb of internal capsule
2	5.83	−6, −20, −4	Midbrain
1	5.82	−2, −37, −26	Pons
1	5.82	−1, −36, −25	Pons
1	5.82	−2, −35, −23	Pons
1	5.82	−27, 4, 23	Superior corona radiata
1	5.78	−17, 0, 11	Knee/posterior limb of internal capsule
1	5.64	−4, −25, −3	Midbrain
1	5.63	−3, −27, −4	Midbrain

Regions with significantly stronger structural disconnection in patients with language impairments (*n* = 17) than in patients without language impairments (*n* = 84). Significance was assessed on the voxel-level with a permutation test with 5000 random permutations of the design matrix. Anatomical labels are based on Johns Hopkins University atlas (JHU) distributed with MRIcron. All regions are located on the left side of the brain.

### Disconnection mapping

**Figure 1:**
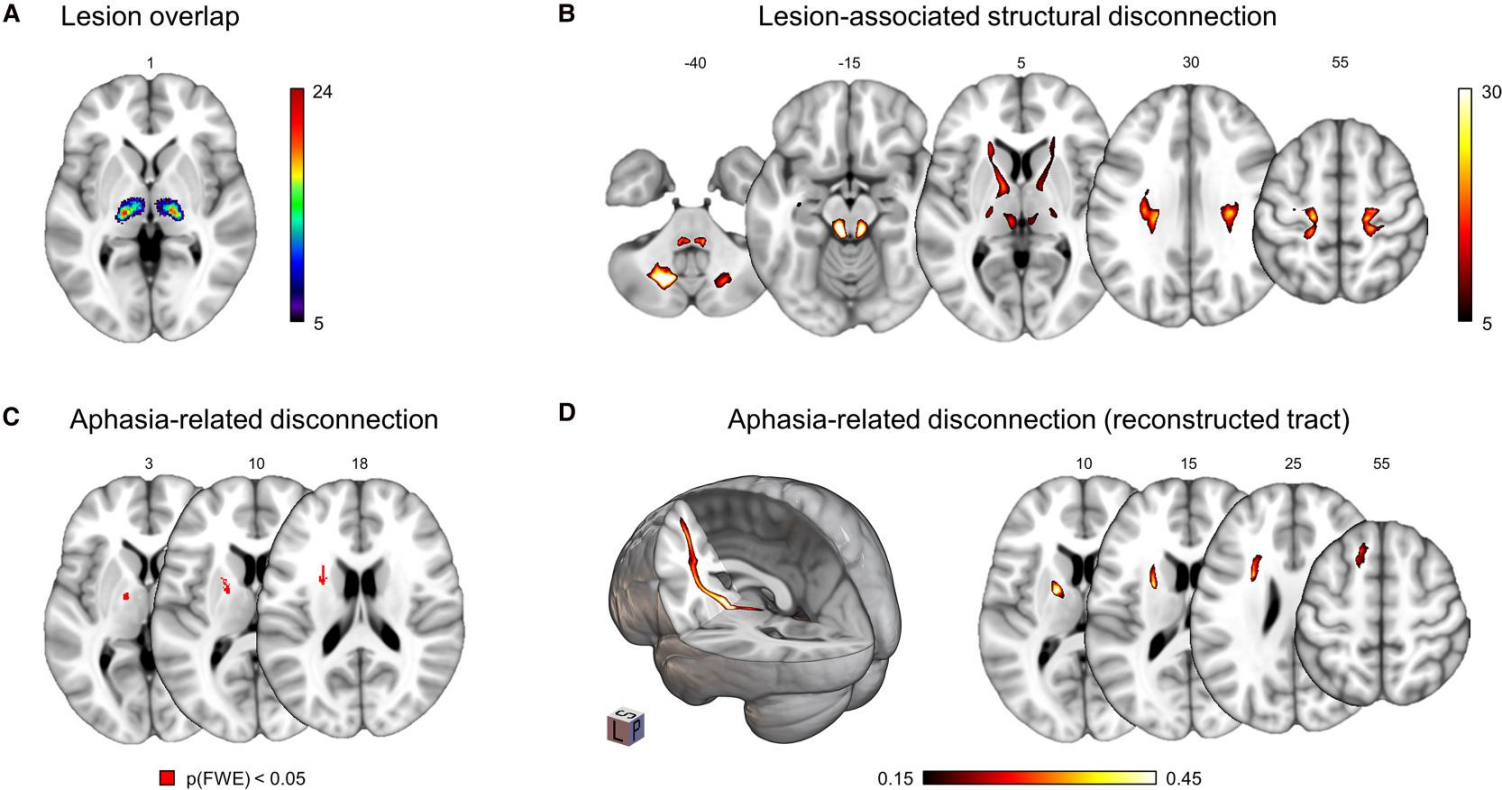
**Results.** (**A**) the overlap of the lesions from all 101 patients which were used to calculate lesion-associated disconnection shown in panel (**B**) as a group overlay. Colour indicates number of patients with a lesion/disconnection per voxel. (**C**) Associations between structural disconnection and thalamic aphasia (17 patients with language impairments versus 84 patients without language impairments, permutation test with 5000 random permutations, *P*(FWE) < 0.05 on the voxel-level, 5-mm maximum intensity projection for display purposes). The significant voxels in the anterior limb of the internal capsule were used as a seed for diffusion tensor imaging (DTI) fibre tractography shown in panel (**D**) to reconstruct the associated tract based on DTI MRI from 25 healthy participants (colour encodes relative fibre count). Coordinates on the axial slices refer to Montreal Neurological Institute (MNI) space in mm. Left hemisphere is shown left. Abbreviations: *P*(FWE): family-wise error corrected *P*-value, L: left, S: superior, P: posterior.

**Figure 2:**
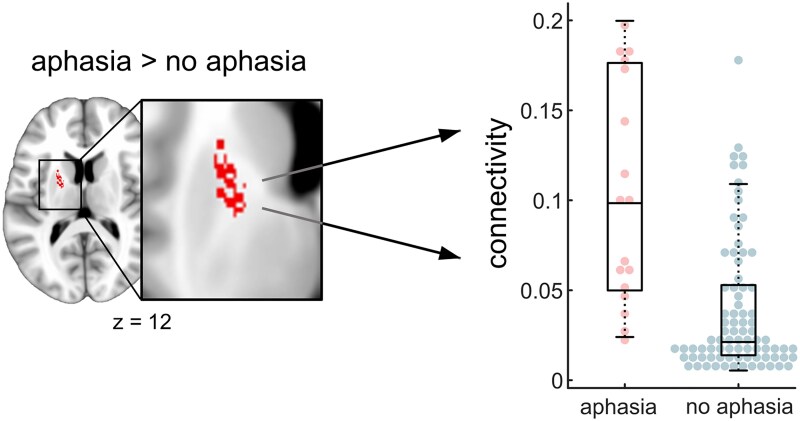
**Disconnection in patients with and without aphasia.** All significant voxels from the main analysis (aphasia-related disconnection, see Fig. 1C and left side of this figure, permutation test with 5000 random permutations, *P*(FWE) < 0.05 on the voxel-level, 5-mm maximum intensity projection for display purposes), were used to extract individual disconnection values (relative fibre counts). These individual values are plotted on the right side of the figure for patients with (left in boxplot, red) and without (right in boxplot, blue) aphasia. Central marks of the boxes represent the median value, the edges are the 25th and 75th percentiles, and the whiskers extend to the most extreme data points not more than 150% the interquartile range beyond the boxes. Filled circles represent individual data points. Coordinate on the axial slices refers to Montreal Neurological Institute (MNI) space in millimetre.

Lesions were localized in the territory of the posterior cerebral artery in both thalami, with a maximum overlap in the left ventral lateral nucleus (Fig. 1A). We performed probabilistic fibre tracking informed by the individual lesion masks to obtain individual disconnection maps. On the group level, these maps covered the internal capsule, the corona radiata, the posterior brain stem and parts of the cerebellar white matter bilaterally (Fig. 1B). We then analysed disconnection–behaviour relationships using a voxelwise permutation test (*P*(FWE) < 0.05 on the voxel-level based on 5000 random permutations). We found a significant association between the individual disconnection profiles and presence of language impairments in voxels located in the anterior limb of the left internal capsule (125 voxels), the left midbrain (7 voxels) and left pons (3 voxels) (Fig. 1C, Fig. 2 and Table 1). When repeating this analysis with right-sided disorders of movement included as an additional covariate of no interest, we obtained nearly identical results (dice similarity coefficient = 0.96). While individual disconnection maps may have terminated in a cortical region, the comparison at the group level did not result in a continuous thalamo-cortical tract. This was likely due to the inherent inter-individual variability of structural connectivity in the normative data and the relatively small sample size. To reconstruct the complete tract and the cortical area it connects to, we performed a second probabilistic fibre tracking seeded from all significant voxels of the first analysis (Fig. 1D). This revealed a tract between the left mediodorsal thalamic nucleus and the left superior frontal gyrus (dorsomedial prefrontal cortex; MNI coordinate in mm: *x* = −13, *y* = 23, and *z* = 62 mm) via the anterior limb and knee of the internal capsule and the anterior corona radiata. This tract belongs to the anterior thalamic radiation which connects the mediodorsal thalamic nucleus with the prefrontal cortex.^16^ The reconstructed thalamic tract also extended caudally via brainstem to the left cerebellum. This was still the case when all infratentorial voxels were ignored and probabilistic fibre tracking was exclusively seeded from the internal capsule.

**Figure 3:**
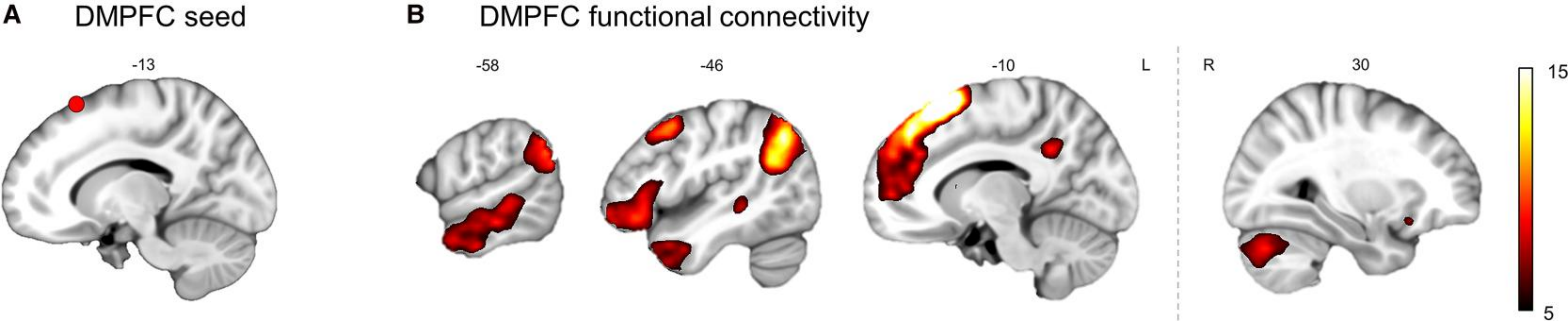
**DMPFC functional connectivity.** Panel (**A**) shows the cortical termination of the aphasia-related thalamo-cortical tract represented by a 6-mm sphere in the dorsomedial prefrontal cortex (DMPFC, red). Panel (**B**) shows the resting-state functional connectivity pattern of this seed in the dorsomedial prefrontal cortex based on resting-state functional MRI data from 65 healthy participants (one-sample permutation test with 5000 random permutations, *P*(FWE) < 0.05 on the voxel-level). Colour indicates *t* value. Coordinates on the sagittal slices refer to MNI space in mm. *P*(FWE): family-wise error corrected *P*-value, L: left, R: right.

In a further exploratory analysis, we followed up the language impairment-related fibre terminations in the dorsomedial prefrontal cortex by means of a seed-based resting-state functional connectivity analysis in the same normative sample as in our previous study.^9^ We were able to demonstrate functional connectivity of the domain-general dorsomedial prefrontal cortex (DMPFC) seed (Fig. 3A) to bilateral prefrontal, cingulate and bilateral inferior parietal cortex, to left hemisphere inferior frontal and lateral temporal cortex, as well as the right cerebellum (Fig. 3B, Supplementary Table 2). When overlaying this result with the main result of our previous study, we found that the language impairment-related pattern of thalamic functional connectivity and DMPFC functional connectivity contained several overlapping regions (Supplementary Fig. 1). These were regions in the bilateral superior frontal gyri (dorsomedial and ventromedial prefrontal cortex), the left middle frontal gyrus (dorsolateral prefrontal cortex), the left inferior frontal gyrus (triangular and orbital parts), the left inferior parietal lobe (angular gyrus) and the lateral posterior temporal cortex (middle and inferior temporal gyrus).

To demonstrate the specificity of the method, disconnection from symptom mapping was repeated for right-sided disorders of movement. Voxels significantly associated with this symptom resembled the left corticospinal tract (Supplementary Fig. 2).

## Discussion

In this observational study, we reanalysed data of a large retrospective cohort of 101 thalamic stroke patients using structural disconnection to symptom mapping. This allowed us to establish a structure–function relationship between thalamic connectivity and thalamic aphasia. In the following, we will discuss our results in light of previous studies that first describe thalamic structural connectivity with language and domain-general brain regions and second relate this connectivity to language functions in particular and cognitive behaviour in general. Third, we extend the discussion on how a dorsomedial prefrontal-thalamic pathway may engage in signalling in distributed networks that concertedly interact to support language processing.

Our main result was that thalamic lesions that cause language impairments are associated with the disconnection of fibres that travel in the anterior limb and knee of the left internal capsule. Even after controlling for right-sided disorders of movement as potential behavioural confounders, this result remained unchanged, supporting the specificity of our finding for the syndrome of interest. The identified fibres constitute a pathway within the anterior thalamic radiation (Fig. 1C). The internal capsule is the primary pathway that connects the thalamus with the cortex. While the anterior limb and knee contain fibres that connect the anterior and mediodorsal nuclei with the prefrontal cortex, the posterior limb consists of the pyramidal tract, but also pathways to the temporoparietal cortex.^16^ Within the anterior limb, separate segments carry fibres to different cortical areas. In the main, dorsally located part of the anterior limb, one pathway connects to the dorsal prefrontal cortices.^25^ This is in line with our secondary result in which we reconstruct a tract that extends to the DMPFC but to no other parts of the language network (Fig. 1D). In contrast, other studies demonstrated structural connectivity of the language-related left inferior frontal cortex (Broca’s area) with ventral anterior nuclei and pulvinar. These fibres were also reported to travel through the anterior limb of the internal capsule.^26^ In parcellation studies^16,27^ and in one lesion study that examined patients with aphasia after left temporal stroke,^28^ the temporal cortex was structurally connected along the posterior internal capsule to the pulvinar and mediodorsal thalamic nucleus. While previous studies typically used seeds for connectivity analysis located in a single region of interest, our study employed spatially variable individual lesion maps. Due to the less frequent involvement of the anterior thalamic nuclei and pulvinar (Fig. 1A), connectivity from these regions may not have been sufficiently disrupted to reconstruct pathways to language-related cortices.

For the first time, we provide empirical evidence that relates language impairments after thalamic stroke to disconnection of fibres in the left anterior limb of the internal capsule. This fits well with case studies of patients with subcortical aphasia after lesions of the anterior limb of the left internal capsule, while lesions in the posterior limb typically caused contralateral disorders of movement and sensory impairments.^11,12^ Beyond this lesion evidence, associations between language abilities and diffusion MRI-based parameters of the internal capsule and anterior thalamic radiation tract integrity also support the idea that a left prefrontal-thalamic pathway contributes to language processing. Alterations in left prefrontal-thalamic connectivity, for example, have been shown to be linked to verbal comprehension.^29^ For aphasic stroke caused by non-thalamic lesions, it was demonstrated that diminished left anterior thalamic radiation tract integrity relates to impaired naming abilities^30^ and the severity of semantic deficits.^31^ This also suggests that in the earlier case studies of subcortical aphasia, which described lesions in the anterior limb of the left internal capsule,^11,12^ a disconnection of the thalamus might have caused the language impairments. It is important to note that evidence also exists for the functional relevance of prefrontal-thalamic pathways for cognition in general. Based on lesion studies, lesions of the anterior limb of the internal capsule and the anterior thalamic radiation cause attentional, memory and executive deficits,^32^ including in patients diagnosed with aphasia.^33^ Similarly, in patients with schizophrenia, reduced mediodorsal thalamus—prefrontal structural connectivity relates to executive deficits.^34^ These patients may also show language impairments, which are typically described as disorganized and incoherent verbal output, a phenotype also observed in patients with thalamic aphasias.^35^ To conclude, the functional contribution of a left hemisphere prefrontal-thalamic pathway to language is supported by our results and previous case reports. However, because no standardized language and cognitive tests were performed, it remains unclear whether the reported impairments were attributable to actual aphasic symptoms and/or an epiphenomenon of undetected attentional, memory and executive deficits. And whilst these deficits may also coexist,^10,36^ other studies demonstrated that language impairments can be observed independently of executive deficits after lesions in the left thalamus^37^ and anterior limb of the internal capsule^11^ and may thus be distinct in nature. Ultimately, though, it must be noted that the language-specificity of the result is limited for this reason.

Our tractography result raises the question of how altered signalling between the left mediodorsal thalamic nucleus and the dorsomedial prefrontal cortex causes language impairments. Because no specific tasks or imaging was performed, we cannot answer this question based on our own data. Instead, we base this part of the discussion about the potential functional relevance of the identified thalamo-prefrontal pathway on the literature. To this end, we will reflect on (i) the role of the prefrontal cortex for cognitive control and (ii) its local implementation through contextually-weighted representations of task rules and goals maintained by prefrontal-thalamic signalling. We will then integrate these findings with the perspective that (iii) language necessitates a meticulous integration in distributed language networks orchestrated by domain-general networks.

The prefrontal cortices contribute to the adaptive control of behaviour towards an intended goal (i.e. cognitive control). This domain-general ability involves a prefrontal generation of representations of task rules or goals that help the flexible selection of distributed sets of active neurons in order to complete a task or achieve a desired behaviour.^38,39^ In theory, these control signals regulate the processing in distributed networks through an enhancement or suppression of neural activity and synchronization between neurons. This prioritizes information to be processed and flexibly shapes its flow in cortical networks that are built in accordance with task rules or goals to implement a specific function.^39,40^

Bidirectional signalling between the prefrontal cortex and the mediodorsal thalamus has been demonstrated to weight local representations of current task rules or goals by amplifying relevant prefrontal synaptic interactions and suppressing irrelevant ones.^41,42^ In this way, the mediodorsal thalamus has been proposed to dynamically select prefrontal representations which then optimally change network parameters (i.e. tune neural interactions) to effectively assemble task-relevant functional cortical networks.^43,44^ Based on healthy fMRI data, a recent study confirmed that thalamic activity best explains different patterns of task-related cortical activity linked to the performance of variable cognitive functions, including language.

This taps into the concept that language is realized in distributed networks. Different aspects of language (e.g. representation of a sound, word or its meaning) or language functions (e.g. comprehension, production) are encoded in spatially distinct patterns of temporally coordinated neural activity across multiple brain regions that flexibly assemble into networks. These language-related representations have been suggested to be controlled by domain-general networks, including the prefrontal cortex.^2,3,45,46^ This interaction is reflected, for example, in one study showing that a part of the domain-general network functionally couples with the left hemisphere language network.^47^ Interestingly, the left anterior lateral subnetwork described in the study (cf. Gordon *et al*. 2020, Fig. 3F) closely resembles the pattern of thalamic functional connectivity we describe in our previous work.^9^ The same pattern is also found in our exploratory functional connectivity analysis seeded from the region in the DMPFC to which the thalamus is structurally connected (Fig. 3). In another study, the idea that language involves a co-activation of brain regions was operationalized in a task that required the representation of different semantic features in order to access meaning.^48^ Contrasting the pattern of fMRI activation in trials where participants successfully accessed meaning compared to trials where they did not, the left thalamus and the DMPFC were most strongly involved in this process. With respect to the role of the prefrontal cortex in language, it was proposed that activation in medial and lateral prefrontal networks controls the goal-directed selection of semantic representations in the language network.^49^ Together, these studies prompt the assumption that for language, prefrontal-thalamic interactions are involved in the dynamic coupling between language and domain-general networks. This may ease the selection of relevant representations of different features and targeted access to meaning. In conjunction with our previous study,^9^ we speculate that the pattern of structural and functional disconnection between the left thalamus, networks for cognitive control and language may result in an inefficient network integration due to inadequately weighted control signals as one potential mechanism of thalamic aphasia.

This study has some limitations. In addition to the general limitations related to the patient cohort and retrospective design addressed in our original publication,^9^ two further limitations must be discussed. First, contrary to our hypothesis, we did not find connectivity to left inferior frontal and temporal cortices. Several methodological constraints may play a role here: Language impairments often coexisted with disorders of movement, thalamic nuclei known to connect left inferior frontal and temporal cortices were rarely lesioned, and the analyses were limited to voxels to be affected in at least five patients. Although the minimum overlap was chosen to increase anatomical validity and disorders of movement were controlled for, it is possible that fibre tracts were missed in this cohort, because disconnection affection in patients with language impairments was too low. The same methodological constraint likely accounts for the lack of a fibre termination in the smaller number of patients with language impairments (*n* = 17), which was readily identified in the larger number of patients with right-sided disorders of movement (*n* = 44) (Supplementary Fig. 2). Thus, our result does not disprove disconnection of fibre tracts to frontal and temporal cortices as another mechanism of thalamic aphasia. On the other hand, our second analysis, although it was informed by the presence of language impairments, may have tracked fibre connections that passed to the seed, but were unrelated to the symptom of interest. In future studies, these limitations could be overcome by including a larger number of patients and the use of an ROI-to-ROI (region of interest) approach. In addition, the application of standardized tests for both language and executive cognitive functions may help to distinguish patients with language impairments secondary to cognitive executive deficits and those that exist independently.^37^ This could reveal whether different patterns of disconnection (e.g. fibre tracts to domain-general prefrontal versus fronto-temporal language networks) specifically contribute to different phenotypes of thalamic language and cognitive impairment.

Second, beyond the large cluster in the anterior limb of the internal capsule, significant language-related disconnection existed in single voxels in the brainstem. Secondary tract reconstruction suggested that fibres travel caudally to the red nucleus and along the superior cerebellar peduncle to reach the left cerebellar dentate nucleus. To our knowledge, no continuous fibre tract is known to connect the internal capsule through the mediodorsal thalamus to reach the cerebellum. As tract reconstruction was seeded in the internal capsule, mediodorsal thalamus connectivity along the anterior thalamic radiation is an anatomically plausible result. For language, the caudally directed tract might rather be a methodological artefact attributable to the non-decussating dentato-rubro-thalamic tract involved in motor control. In contrast to the decussating fibres of this tract that terminate in ventral lateral and anterior sensorimotor thalamic nuclei, non-decussating fibres connect to the more medially located centromedian nucleus.^50^ Because of the close proximity of the centromedian nucleus to the mediodorsal nucleus, nearby fibres could have potentially been traced by the probabilistic fibre tracking algorithm. If language-related mediodorsal thalamus cerebellar connectivity were no artefact, it would have to be discussed within a wider model of subcortical contributions to language,^7,28^ although connectivity to the right cerebellum rather than the left side would be expected.

To summarize, our study provides an anatomical basis for a thalamic involvement in language. Our result suggests that interrupted signalling along the left anterior thalamic radiation to the dorsomedial prefrontal cortex is one potential mechanism of thalamic aphasia. This also suggests that in some historical reports of subcortical aphasia, which described lesions in the anterior limb of the left internal capsule, a disconnection of the thalamus might have caused the language impairments. Given its exemplary nature for a subcortical contribution to language, we propose that a thalamic-prefrontal pathway is a potential gateway for thalamic signals to engage in the interplay between networks for cognitive control and language. Based on concepts of cognitive control and language, we speculate that the functional role of the identified thalamic-prefrontal pathway for language is the weighting of control signals that concert the processing between cognitive control and language networks.

## Supplementary Material

fcaf191_Supplementary_Data

## Data Availability

The data that support our findings (normalized lesions maps, disconnection maps and behavioural data) and the code generated are openly available via FigShare at https://figshare.com/articles/dataset/Thalamic_Aphasia/19154153. This study is reported in accordance with the STROBE checklist.^51^
