# Supplementary material for: Thalamic disconnection from prefrontal cognitive control networks contributes to thalamic aphasia
Source: Brain Commun. 2025 May 16;7(3):fcaf191. doi: 10.1093/braincomms/fcaf191 (PMC12116879; doi:10.1093/braincomms/fcaf191)
Supplement: fcaf191_Supplementary_Data [file fcaf191_supplementary_data.pdf]

Supplementary Table 1

	Dysarthria	Left Motor	Right Motor	Left Sensory	Right Sensory
**Aphasia**	0.41	1.00	**< 0.001**	1.00	0.75
**Dysarthria**		0.29	0.07	0.98	0.29
**Left Motor**			1.00	**< 0.001**	1.00
**Right Motor**				1.00	**< 0.001**
**Left Sensory**					1.00

Supplementary Table 1. Statistical dependence among behavioral variables. Independence between all pairs of behavioral variables was assessed using one-tailed Fisher's exact tests. Reported p-values are uncorrected. Low p-values indicate statistical dependence between the respective variables.

Supplementary Table 2

Cluster size k	Peak t value	Peak coordinate (x, y, z in mm)	Anatomical label (JHU atlas)
1627	10.35	44, -52, -42	Right cerebellum
1	5.37	32, -58, -46	Right cerebellum
2289	11.11	-52, 12, -30	Left pole of middle temporal gyrus
	10.23	-48, -38, -4	Left posterior middle temporal gyrus
	10.06	-64, -20, -12	Left middle temporal gyrus
1	5.41	-44, -2, -44	Left inferior temporal gyrus
12	6.17	6, -52, -40	Right cerebellum
820	9.43	48, 12, -38	Right pole of middle temporal gyrus
	8.65	58, -6, -26	Right middle temporal gyrus
	8.31	54, 0, -34	Right pole of middle temporal gyrus
88	7.02	-22, -76, -28	Left cerebellum
1	5.44	44, -80, -30	Right cerebellum
1651	10.43	-50, 26, 4	Left inferior frontal gyrus pars triangularis
	10.33	-50, 26, -2	Left inferior frontal gyrus pars orbitalis
	9.76	-46, 32, -12	Left inferior frontal gyrus pars opercularis
105	6.70	-4, 34, -10	Left rostral anterior cingulate gyrus
	6.59	-4, 28, -8	Left subcallosal anterior cinglate gyrus
	6.33	-4, 38, -22	Left gyrus rectus
24	7.41	32, 22, -16	Right lateral fronto-orbital gyrus
197	7.16	50, 30, -8	Right inferior frontal gyrus pars orbitalis
	6.46	47, 46, -12	Right middle frontal gyrus (dorsal prefrontal cortex)
8432	30.49	-12, 24, 60	Left superior frontal gyrus (posterior segment)
	15.85	-12, 40, 44	Left superior frontal gyrus (prefrontal cortex)
	11.87	-38, 19, 49	Left middle frontal gyrus (posterior segment)
3	5.52	46, -38, -2	Right posterior middle temporal gyrus
118	8.89	-14, 12, 12	Left caudate nucleus

Cluster size k	Peak t value	Peak coordinate (x, y, z in mm)	Anatomical label (JHU atlas)
44	6.34	16, 14, 8	Right caudate nucleus
12	5.78	10, 58, 6	Right superior frontal gyrus (prefrontal cortex)
1985	16.34	-48, -62, 42	Left angular gyrus
564	11.43	58, -60, 32	Right angular gyrus
486	9.50	-8, -50, 34	Left precuneus
	5.47	8, -52, 32	Right precuneus
50	8.48	-2, -18, 38	Left dorsal anterior cingulate gyrus
21	6.45	38, 24, 40	Right middle frontal gyrus (posterior segment)

Supplementary Table 2. Resting-state functional connectivity of left dorsomedial prefrontal cortex. Functional network of to the grey matter region (MNI coordinate -13 23 62 mm, 6 mm sphere) fibers associated with language impairments were structurally connected to. Results were based on resting-state fMRI data from 65 healthy participants and a permutation test with 5,000 random permutations (p(FWE) < 0.05 on the voxel-level). The table shows up to three peaks in different anatomical regions per cluster. Anatomical labels are based on John Hopkins University atlas (JHU) distributed with MRIcron.

Supplementary Figure 1

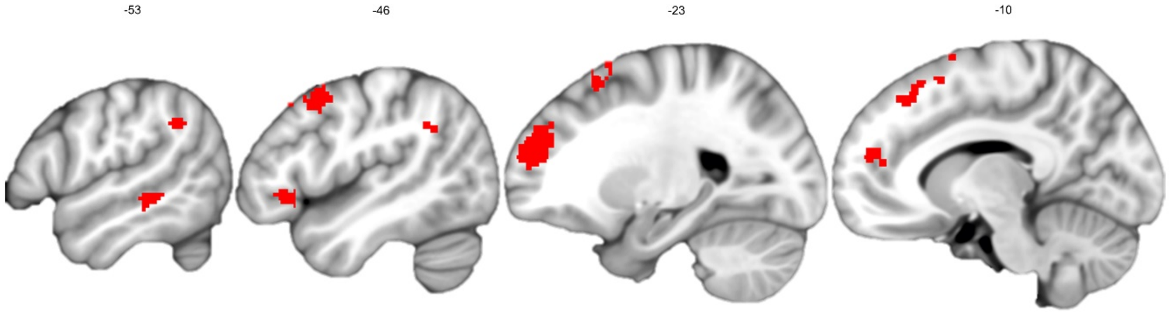

Supplementary Figure 1. Overlap of DMPFC functional connectivity and lesion network connectivity associated with thalamic stroke. This figure shows the overlap between the dorsomedial prefrontal cortex (DMPFC) functional connectivity (c.f. Figure 3B in the manuscript) and lesion network connectivity associated with thalamic aphasia from our prior study (c.f. Figure 4A in Stockert et al., 2023). Coordinates refer to MNI space in mm. All sagittal slices are on the left side of the brain.

Supplementary Figure 2

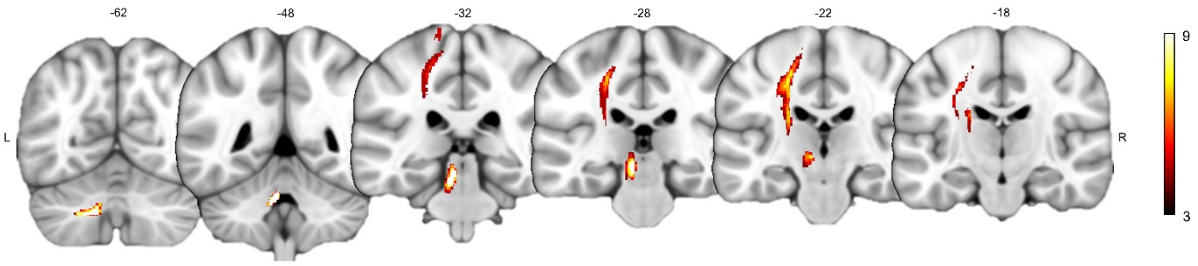

Supplementary Figure 2. Disconnection-to-symptom mapping for right sided disorders of movement. Colored voxels indicate a significant association between structural disconnection and right sided disorders of movement (44 patients with vs. 57 patients without impairment, permutation test with 5,000 random permutations, p(FWE) < 0.05 on the voxel-level). Coordinates on the coronal slices refer to MNI space in mm. Abbreviations: p(FWE): family-wise error corrected p-value, L: left, R: right.

Supplementary References

1. Stockert A, Hormig-Rauber S, Wawrzyniak M, et al. Involvement of Thalamocortical Networks in Patients With Poststroke Thalamic Aphasia. Neurology. 2023;100:e485-e496.
